# Rare Copy Number Deletions Predict Individual Variation in Intelligence

**DOI:** 10.1371/journal.pone.0016339

**Published:** 2011-01-26

**Authors:** Ronald A. Yeo, Steven W. Gangestad, Jingyu Liu, Vince D. Calhoun, Kent E. Hutchison

**Affiliations:** 1 Department of Psychology, University of New Mexico, Albuquerque, New Mexico, United States of America; 2 The Mind Research Network, Albuquerque, New Mexico, United States of America; 3 Department of Electrical and Computer Engineering, University of New Mexico, Albuquerque, New Mexico, United States of America; 4 Department of Psychology and Neuroscience, University of Colorado, Boulder, Colorado, United States of America; University of Utah, United States of America

## Abstract

Phenotypic variation in human intellectual functioning shows substantial heritability, as demonstrated by a long history of behavior genetic studies. Many recent molecular genetic studies have attempted to uncover specific genetic variations responsible for this heritability, but identified effects capture little variance and have proven difficult to replicate. The present study, motivated an interest in “mutation load” emerging from evolutionary perspectives, examined the importance of the number of rare (or infrequent) copy number variations (CNVs), and the total number of base pairs included in such deletions, for psychometric intelligence. Genetic data was collected using the Illumina 1MDuoBeadChip Array from a sample of 202 adult individuals with alcohol dependence, and a subset of these (N = 77) had been administered the Wechsler Abbreviated Scale of Intelligence (WASI). After removing CNV outliers, the impact of rare genetic deletions on psychometric intelligence was investigated in 74 individuals. The total length of the rare deletions significantly and negatively predicted intelligence (r = −.30, p = .01). As prior studies have indicated greater heritability in individuals with relatively higher parental socioeconomic status (SES), we also examined the impact of ethnicity (Anglo/White vs. Other), as a proxy measure of SES; these groups did not differ on any genetic variable. This categorical variable significantly moderated the effect of length of deletions on intelligence, with larger effects being noted in the Anglo/White group. Overall, these results suggest that rare deletions (between 5% and 1% population frequency or less) adversely affect intellectual functioning, and that pleotropic effects might partly account for the association of intelligence with health and mental health status. Significant limitations of this research, including issues of generalizability and CNV measurement, are discussed.

## Introduction

Behavior genetic studies over the past several decades leave little doubt that psychometric intelligence (IQ or *g*) is partially heritable, with estimates varying from 40% to 80% and increasing with age [1], [2]. But what genetic factors play a role? This question, traditionally important, has become of even greater interest in light of documented associations of intelligence with health [3], [4], mortality [5], [6], psychopathology [7], [8], and diverse social outcomes [9], in conjunction with evidence that these relationships are partially or largely due to heritable components of *g* [10]–[13]. The significance of variations in intelligence has also been examined among individuals with alcohol dependence, as lower intelligence as assessed in childhood or in early adulthood predicts greater comorbidity [14], a greater propensity for hangovers [15], greater mortality from alcohol-related health problems [16], and poor treatment outcomes [17].

Beginning nearly two decades ago, behavior geneticists increasingly searched for individual allelic variations associated with *g*. Despite a good number of candidate gene and SNP (single nucleotide polymorphism) association studies, very little progress has been made—so little, in fact, that a recent review led the authors to conclude, “it is not possible confidently yet to name one genetic locus unequivocally associated with the quantitative trait of intelligence” ([18], p. 219). It seems clear that no one locus accounts for more than a very small amount of the genetic variation in *g*.

If no one locus accounts for much variation in g, a likely possibility is that *g* is massively polymorphic. Recent studies of stature and personality variations (e.g., neuroticism) show that many loci contribute effects, with no one locus accounting for more than 1% of the variation [19], [20]. A reasonable conjecture is that much of this variation arises due to mutation, with frequencies of secondary functional alleles being low. As mutations at many loci may affect expression of high-level phenotypic features (whose development is affected by many individual pathways), such features (e.g., stature) may be affected by many genetic variations.

Researchers have proposed that *g* is just such a phenotypic feature [21]–[25]. In part, this conjecture emerged from findings that *g* is associated with developmental instability, as assessed by composite measures of random variations in bodily symmetry. Meta-analyses show human developmental instability is associated with reduced psychometric intelligence and neurodevelopmental disorders [26], [27]. More generally, developmental instability is associated with worse health outcomes, illness, and fetal stresses [27], which could be due to deleterious effects of widespread mutation and explain links of intelligence with mortality. In fact, however, mutations could affect *g* independent of developmental instability too; a great many genes are expressed in the brain [28], and genome-wide pleiotropy is substantial [29]–[31].

One problem with testing these ideas by examining the association between the genome-wide mutation load and *g* (or any other phenotypic trait) is that, technologically, it is not possible to measure genome-wide mutations at this time. But recent advances in genomic studies have led to the discovery of one potentially important class of rare variations that *can* be assessed with current technology, copy number variations (CNVs). It was once thought that the “normal” human genome could be defined by a shared reference genomic structure, one specifying all single nucleotide sites. In its extreme form, this view implies that all genetic variation between any two (“normal”) individuals consist merely of the aggregate of base differences across all 3 billion or so single nucleotide sites. Geneticists have long recognized the existence of exceptions—insertions, deletions, or inversion of long chromosomal segments in individual genomes. Recent discoveries, however, show that “exceptions” are anything but unusual [32], [33]. A substantial portion of the genome is subject to “copy number variation”—differences across individuals in number of copies of a chromosomal segment at least 1000 bases long (i.e., rather than possessing 2 copies of the segment, possessing 0, 1, 3 or more). Thus far, several thousand such sequences have been found in the human genome, comprising about 30% of it [34]. Variation across individuals, then, consists not only of differences at single nucleotide sites, but also in number of copies of particular DNA strands.

Ultimately, CNVs originate as mutational events [34]. Like most point mutations, the majority are probably mildly deleterious. That is particularly true of deletions, and especially ones maintained at low frequencies (<5%, [35]); on average, insertions appear to be less deleterious, and some CNVs may exist at high relative frequencies due to relaxed selection. Also similar to point mutations, CNVs may persist in a lineage for many generations despite selection against them. Compared to point mutations, however, CNVs have a much higher de novo mutation rate, 10–1000 times as great [34], [36], which means that, for a given strength of selection against them, their equilibrium frequency in the population will be greater. Moreover, whereas point mutations affect a single nucleotide base, CNVs affect many, with multiple genes sometimes affected by a single CNV. As a result, CNVs may account for more total inter-individual genetic variation than single nucleotide variants combined [37]. Unlike point mutations, genome-wide CNVs can be measured in population studies using a number of methods, including SNP microarrays. A recent population study found that CNVs larger than 500 Kb occur in 5–10% of the population, with 1–2% possessing one or more CNV 1 Mb or larger. On average, individuals possessed 3–7 CNVs, very conservatively estimated in this study [35].

Recent studies have revealed an elevated incidence of rare deletions in schizophrenia, autism, and mental retardation [38]. Some studies have linked CNVs in specific locations to phenotypic variation. For example, Bi et al. [39] have found submicroscopic duplications in 17p13.3 involving LIS1 are linked with structural brain abnormalities and developmental delay. Other studies have noted the presence of widespread, rare abnormalities in association with such diagnoses as autism [40], schizophrenia [41], and bipolar disorder [42]. Some of these disorders covary with intelligence, with, e.g., 92% of the phenotypic covariation between schizophrenia and intelligence reflecting genetic covariation (e.g., [43]). In addition, neurodevelopmental disorders such as schizophrenia are characterized by increased developmental instability [44].

Not all diseases are associated with specific CNVs [45]. There may be systematic reasons why CNVs affect psychological traits in particular. CNVs are not randomly distributed throughout the chromosome, as they tend to be over-represented in “hot-spots,” regions where high rates of segmental duplication (in effect, insertions that have been driven to near-fixation) lead to more frequent non-allelic homologous recombination (e.g., [46]). These regions of segmental duplications, in turn, tend to have evolved relatively recently. Though comprising only about 5% of the human genome, for instance, segmental duplications account for more divergent evolution between chimpanzees and humans than all single base-pair changes combined [47]. These regions are likely to play critical roles in the development and expression of many traits derived in the human lineage, phenotypically distinguishing us from close relatives. Not surprisingly, then, widespread segmental duplications contain genes involved in neuronal development or expressed in neural tissues, perhaps central to human-specific cognitive features (e.g., [48]–[50]). As a result, CNVs (particularly large, rare deletions) may more strongly affect these same features, thereby influencing, e.g., *g*.

In the current study, we sought to test the prediction that rare CNVs covary with *g* across its normal range. The definition of “rare” is somewhat arbitrary and different researchers have used different criteria. In studies of schizophrenia, 1% frequency (percentage of a sample with the variant) has often been used as a cutoff for rare [41]. Studies have used a 5% cutoff to separate “common” from ”non-common” variants [51]. In some parlances, variants with 5% of less representation include those that are “rare” as well as ones that are of “low frequency.” More common variants are expected to have less severe phenotypic effects [35], which could include an effect on *g*, prompting our decision to use the 5% figure. Hence, variants we aggregated include those that qualify as both rare and infrequent (though, we note, we also examined associations using more strict cutoffs of 3% and 1%; see Results). We measured CNV deletions in two ways: total number of CNV deletions and total length (in bases) of CNV deletions (where the latter measure weights each CNV by its length). Given recent findings that heritability of intelligence may be greater in children [52] and adolescents [53] from social backgrounds with relatively greater parental socioeconomic status, we also undertook preliminary analysis of this issue. Though we made no prediction regarding rare insertions, for completeness we also examined associations between *g* and these variants.

## Materials and Methods

### Subjects

Subjects were recruited from the general community for a study designed to investigate genetic correlates of alcohol dependence and related phenotypes such as intelligence (14). Inclusion criteria were (1) age between 21 and 55; (2) within 21 days of their last drink; (3) drinking levels of more than 14 drinks per week (females) or 21 drinks per week (males) during four consecutive weeks within three months of beginning the study; (4) negative drug screen for opiates, cocaine, or amphetamine; (5) must meet DSM IV criteria for alcohol dependence; and (6) must have a Clinical Institute Withdrawal Assessment (CIWA) [54] score of less than 8, indicating no need for medical detoxification. Alcohol dependence was also measured continuously with the alcohol dependence scale (ADS), a 25-item test that has four subscales tapping loss of behavioral control, obsessive-compulsive drinking style, and psychophysical and pyschoperceptual withdrawal symptoms [55].

### Ethics Statement

All subjects provided written consent. The study was approved by the University of New Mexico Human Research and Review Committee according to principles expressed in the Declaration of Helsinki.

### Genetic Analyses

Participants provided at least 5 ml of saliva into a sterile 50 ml conical centrifuge tube. DNA was then extracted from the sample, purified, and hybridized. Detection of 1,199,187 SNP and CNV markers across the entire genome was performed using the Illumina Human 1 M Duo BeadChip Array according to the manufacturer's directions. We did not analyze DNA from the X and Y chromosomes, reducing the number of SNPs to 1,147,842. The data were further scanned and 10,272 loci with missing measurements were removed. The median distance between adjacent markers was approximately 2.5 kbp.

Details of the series of procedures used to quantify CNVs are described in Chen, Liu, and Calhoun [56]. Briefly, principal component analysis (PCA) was performed to minimize noise effects and remove extraneous sources of variance, including batch effects, as well as variances related to GC percentage. Next, samples were eliminated if they appeared to be outliers as determined by the standard deviation of the Log R Ratio larger than 0.28 (see [57]). The preprocessed data were segmented independently using two methods, the circular binary segmentation (CBS) algorithm implemented in MATLAB and a hidden Markov model (HMM) algorithm implemented in PennCNV [58]. To be counted as a CNV, a segment needed to be identified by both approaches. This is a conservative approach to CNV identification designed to increase reliability of detection. We classified rare CNVs as those occurring in 5% or less of the sample. For both rare deletions and insertions we calculated the total number of CNVs, as well as the total number of base pairs included in each type of abnormality.

### Intelligence Assessment

All participants were administered the vocabulary and matrix reasoning subtests of the Wechsler abbreviated scale of intelligence (WASI; [59]), from which a full scale intelligence quotient (FSIQ) was calculated. The vocabulary test taps verbal/crystallized functioning and the matrix reasoning test taps nonverbal/fluid reasoning. A FSIQ score was derived from these two tests using age-appropriate norms. The average reliability of the FSIQ is 0.93 [59]. The subtests have a mean of 50 and a standard deviation of 10, while FSIQ has a mean of 100 and a standard deviation of 15.

## Results

Genetic data was available for 202 participants, while WASI data were available for only 77. Initial evaluation of CNV numbers was performed on the larger sample, so as to maximize accurate evaluation of the shape of the frequency distributions. Six samples were discarded using Need's criteria, leaving a sample of 196 participants. At total of 13,557 CNVs were detected, 7249 deletions, 6308 insertions (minimum = 10, median = 51, maximum = 560). The observed frequency distribution of CNV number was markedly skewed, as a few participants had extremely high numbers of CNVs. As oversensitivity of CNV detection for these individuals likely led to unrealistically high values [35], we eliminated extreme outliers, ones exceeding Tukey's criterion of the third quartile value plus three times the inter-quartile range [60]. For the participants with WASI data, use of this criteria resulted in discarding 3 cases (4% of the sample), resulting in a final data set of 74 participants (51 male, 23 female). Self-reported ethnicity of the sample was White/Anglo (N = 31, 42%), Latino (N = 26, 35%), Native American (N = 4, 6%), African-American (N = 2, 3%), Asian (N = 1, 1%), and Mixed (N = 9, 12%). One person chose not to report ethnicity. Table 1 provides descriptive statistics on demographic variables, genetic variables, and test performance.

**Table 1:** Descriptive statistics on demographic, genetic, and test data (N = 74).

	Mean	SD	Range
Age	39.89	9.24	22–55
Education (years)	13.42	2.13	8–20
Alcohol Dependence Scale	16.91	7.23	4–43
Number rare deletions	10.95	5.48	1–25
Length rare deletions (bp)	210,618	14,386	8083 - 626,241
Number rare insertions	6.47	9.82	0–63
Length rare insertions	356,238	53,327	0–2,278,718
WASI Full Scale IQ	98.20	13.59	71–135
WASI Vocabulary	47.47	10.68	20–69
WASI Matrix Reasoning	50.13	9.48	29–70

**Figure 1:**
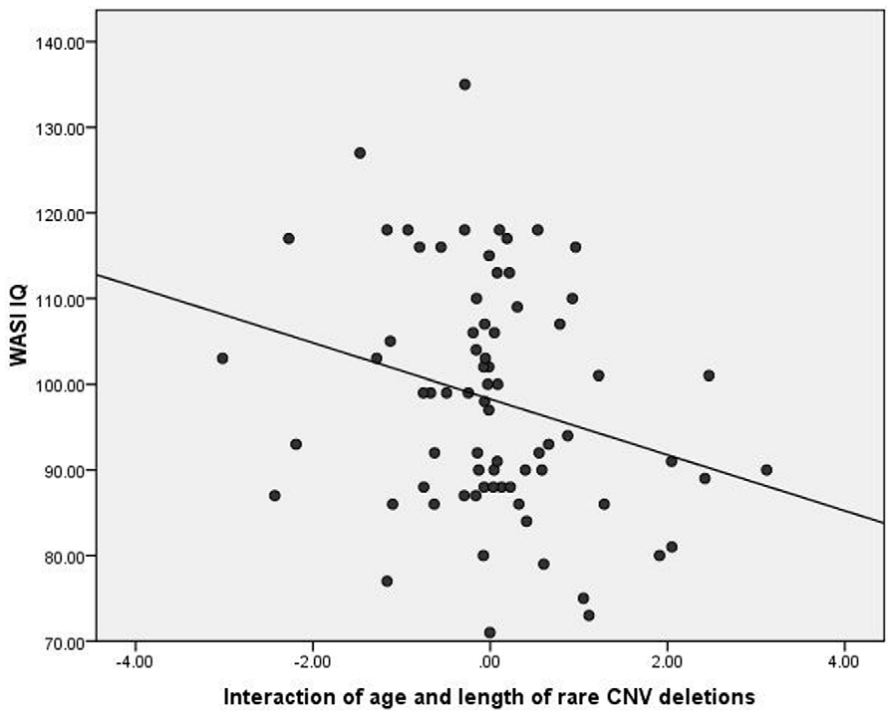
Scatterplot of the relationship between length of total rare deletions (base pairs) and Full Scale Intelligence Quotient (r = −.30, p = .01).

Pearson correlation coefficients between rare CNV variables and intelligence test performance are presented in Table 2. As predicted, the total base pair length of rare deletions negatively and significantly (p = .01) covaried with FSIQ. A trend was noted for the number of rare deletions (p = .08). Figure 1 presents a scatter plot of the relationship between total rare deletion length and FSIQ. Length of deletions was also negatively correlated with the Matrix Reasoning subtest (p = .013), but not the Vocabulary subtest.

**Table 2:** Pearson correlation coefficients (and significance levels) between Wechsler Abbreviated Scale of Intelligence variables and the number and total size (in base pairs) of rare deletions and insertions (N = 74).

	Full Scale IQ	Vocabulary	Matrix Reasoning
Length rare deletions	−.30 (p = .01)	−.16 (ns)	−.29 (p = .013)
Number rare deletions	.21 (p = .08)	−.12 (ns)	−.16 (ns)
Length rare insertions	−.03 (ns)	.02 (ns)	−.08 (ns)
Number rare insertions	−.07 (ns)	−.07 (ns)	−.07 (ns)

Adding total ADS score as a covariate did not alter these findings, and total ADS did not correlate with any CNV variable (all *p* values >0.17). Hence, these findings are not driven by an association between FSIQ and severity of alcohol dependence. One might also wonder, however, whether they are driven by duration of alcohol abuse. If this were the case, one would anticipate correlations between age, as a proxy measure for duration of alcohol abuse, and the three cognitive measures. None of these correlations was significant (all *p* values greater than 0.58).

For completeness, we report that rare insertion variables were not related to intellectual ability (Table 2).

We performed regression analyses to examine possible moderating effects of sex and ethnicity, where ethnicity was simply coded as “Anglo/White” and “Other Ethnicity”. We were not interested in ethnicity per se, but rather were interested in possible moderating effects of parental socioeconomic status (SES), for which ethnicity might serve as a proxy variable. Prior research has found that parental SES affects heritability estimates for intelligence. Data on parental SES were not available for this sample. In New Mexico, where the sample was collected, individuals of other ethnicity (e.g., Latino, Native American), on average, come from lower SES backgrounds than those of Anglo/White ethnicity.

In these regression analyses (run on PASW [formerly SPSS] 17.0 GLM univariate), FSIQ served as the criterion variable. Predictors entered were (1) sex, (2) ethnicity, (3) rare deletion length, (4) rare insertion length, and (5) interactions of each of the CNV variables with sex and ethnicity. Results revealed a main effect of deletions, partial eta  = 0.38, F(1,63)  = 11.00, p = 0.002. This effect was significantly moderated by ethnicity, partial eta  = 0.39, F(1,63)  = 11.48, p = 0.001. Ethnicity also had a main effect independent of deletions and insertions, β = 0.53 F(1,63) = 24.36, p<.001. No other effect was statistically robust, all p>.28.

To evaluate the robustness of the effect across varying definitions of infrequent or rare deletion, we repeated this regression analysis twice, each time substituting a measure of CNV length based on a different, more stringent definition of rare CNVs,. Specifically, we calculated length using criteria for rare CNVs of “≤3%” and “≤1%”. In each analysis, the effect of deletion length was statistically significant, with the ≤3% definition yielding slightly stronger effects than our original <5% definition, and the ≤1% definition yielding slightly weaker effects: partial eta  = .41, p<.001, and. 31, p = .013, respectively. Both analyses also yielded the significant interaction between deletion length and ethnicity. In sum, then, the effect of infrequent deletions in this sample is not peculiar to a criterion of a relative frequency of 5% or less.

**Figure 2:**
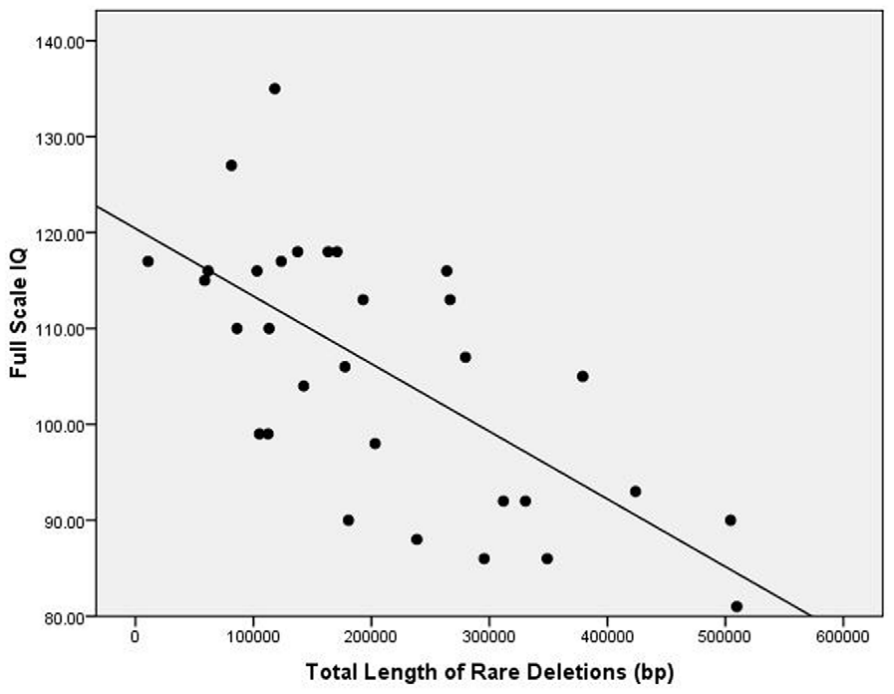
Scatterplot of the relationship between length of total rare deletions (base pairs) and Full Scale Intelligence Quotient in the Anglo/White group (r = −.68, p<.001).

To examine the nature of the deletion x ethnicity interactions, we separately computed correlations for the Anglo/White and Other groups. As can be seen in Table 3, much stronger relationships between deletions and intelligence test performance were observed in the Anglo/White ethnic category. Figure 2 shows a scatterplot of the relationship between length of rare deletions and Full Scale IQ in the Anglo/White group.

**Table 3:** Pearson correlation coefficients (and significance levels) between Wechsler Abbreviated Scale of Intelligence variables and the number and total size (in base pairs) of rare deletions in Anglo/White (N = 31) vs. Other (N = 42) ethnic group categories.

Group		Full Scale IQ	Vocabulary	Matrix Reasoning
Anglo/White	Length rare deletions	−.68 (p<.001)	−.55 (p = .001)	−.53 (p = .002)
	Number rare deletions	−.34 (p = .06)	−.21 (ns)	−.26 (ns)
Other	Length rare deletions	−.11 (ns)	.04 (ns)	−.18 (ns)
	Number rare deletions	−.06 (ns)	−.02 (ns)	−.05 (ns)

We computed the residual variance in FSIQ with variation associated with deletion length in each group was removed. There was no difference in residual variance across groups, p = 0.41. Hence, the Anglo/White group had significantly more variance in FSIQ associated with deletions, but there was no significant difference in the amount of variance in IQ not associated with deletions.

No significant differences related to ethnicity category were found in independent samples t-tests (all p values >0.3) for any of the four genetic variables (number and length of rare insertions and deletions). We emphasize, then, that, while rare CNV deletion lengths covary differently for the two ethnic categories (possibly due to differences in parental SES), main effects of CNVs do not contribute to differences in cognitive performance of the two groups.

## Discussion

The greater the size of rare and infrequent deletions, as represented by the number of base pairs lost, the lower an individual's psychometric intelligence. In contrast to SNP effects investigated in prior studies, the current effect size is substantial, accounting for 9% of the phenotypic variance in the full sample and 45% in the Anglo/White sub-sample.

There are several important implications of this finding. Before discussing them, however, we consider the generalizability of the current results. Our sample consisted of individuals with alcohol dependence. Do associations between intelligence and rare deletions exist in other populations? Only replication in other samples of healthy controls and clinical groups will provide a clear answer to this critical question. At present, however, no specific observations suggest the relationship is unique to the current sample. First, overall WASI performance is solidly in the average range, only very slightly below the mean level of the general population. Second, controlling for a measure of alcohol dependence (the ADS) did not diminish the association. Third, the greater genetic effect in the Anglo/White sample is consistent with prior studies in healthy children and adolescents showing greater heritability in higher SES groups [52], [53], [61]. In light of these findings, we have no reason to believe that the associations we observed will not generalize to healthy populations.

The measure of CNV deletions we used aggregates many different deletions. The genetic effect demonstrated here is different in kind than that revealed by studies of individual SNPs or large aggregations of SNPs (e.g., [20]). The rare or infrequent deletions we tabulated were scattered across the genome, and by definition occur at a given locus in less than 5% of our sample (that is, 9 of 196 individuals). But in fact, most had fewer. In that sample, we detected 3363 distinct rare or infrequent CNVs (that is, ones at different sites). Of those, nearly 80% were detected in fewer than ∼1% (1 or 2). A mere 3% were detected in more than 3% of individuals (7–9). The mean, median, and mode of the percentage detected were ∼1%, 5%, and 5%, respectively. And these values include insertions; deletions tended to represented in rarer CNVs than insertions (see also [35]).

These facts have two important implications. First, different people have different deletions. Indeed, given the distribution of infrequent deletions, it stands to reason that the large majority of random pairs of individuals share *none* of these deletions. And for any two individuals possessing even a handful of them, the probably that they share precisely the same ones is vanishingly small.

Second and relatedly, it must then be the case that many different individual CNVs have common effects on intellectual functioning, with no one CNV deletion possessing any more than a very small effect. (Indeed, though total CNV deletions predict FSIQ much better than any SNP does, any *particular* deletion may do no better than any individual SNP). Our sample is of insufficient size to examine the effects of many individual CNV deletions. But with 3364 individual rare variations possible, it seems unlikely that only a small subset carries the effect we observe. The reason is simple: Each CNV deletion can possibly account for only a tiny amount of total variation in the aggregate measure of infrequent deletions. Were it the case that only a small subset (say <10%—still, well over 100) had reliable associations, their variation accounted for would be overwhelmed by the variation in the remaining non-relating deletions (say >90%), leaving a very small amount of variation accounted for. Hence, the only plausible view is that a sizeable number of infrequent deletions covary with IQ.

Though the total number of possible deletions is too many for us to examine all, we *did* examine a small subset in analyses not detailed in this report. Itsara et al. [35] listed CNV deletions in 13 different chromosomal regions that previous work has shown are associated with psychological disorders (schizophrenia, autism, mental retardation). Of our sample of 74, a total of 14 individuals had a deletion in one of these regions. (Five regions accounted for all 14 cases.) We asked whether, after controlling for ethnicity and sex, the IQ scores of these 14 significantly differed from those of the remainder of the sample. They did not, F(1, 68) <1, p = .50. From this analysis, we cannot conclude that deletions in these regions do *not* have effects; they may well have small effects. Rather, the point is that, not surprisingly, this small subset previously found to predict psychological outcomes do not drive the association of our rare deletion composite and *g*. (Details of results available from the authors by request.)

We did not screen CNVs to have any particular function or be located in any particular place in the genome. Why, then, would deletions in many regions of the genome affect intellectual functioning? We discuss two important considerations.

First, there may exist massive pleiotropy [29]. A given gene likely contributes to many different metabolic and developmental pathways, so loss of genetic material at a particular locus may have widespread effects. Hence, even genes with primary functions pertaining to outcomes other (or broader) than intellectual functioning may affect intellectual functioning. Relatedly, a great many genes contribute to brain function; in the mouse, as many as 80% of all genes are expressed in the brain [28]. As a result, randomly placed deletions may be more apt to have a deleterious effect on brain function than not.

The association of rare deletions with intelligence may, in this context, shed light on why lower intelligence predicts more comorbid health and mental health problems: holes in the genome may have widespread consequences, especially for brain function. For example, two disorders commonly comorbid with alcohol problems are the “externalizing” disorders of ADHD and antisocial behavior. Each of these disorders is associated with reduced intelligence [62] and its phenotypic correlation with each disorder is completely accounted for by genetic covariance [12], [63]. The current results lead to the prediction that these other disorders would also be associated with greater rare deletions. For another example outside the substance abuse field, consider the greater risk for Alzheimer's disease conferred by lower premorbid intelligence [64], [65]. Though greater intelligence might be protective in innumerable ways related to lifestyle choices, greater mutation load might also lead to greater metabolic stress and reduced capacity for maintenance of brain integrity [66].

Second, it is possible that genetic material prone to deletion is more associated with intellectual functioning than randomly selected genetic material in the human genome. One process generating both deletions and insertions, nonallelic homologous recombination (NAHR), tends to produce CNVs near “hot-spots” [46]. These hotspots themselves tend to be rich in segmental duplication, repeated segments that have evolved through positive selection for particular duplications. Segmental duplications are much more common in primates than other mammals [47]. As recent human evolution may have involved duplication more than alteration at single nucleosides, in addition to the fact that brain size and function evolved in hominines, one might well expect that relatively more material duplicated in the genome is expressed in the human brain; some research supports this expectation [50]. Another process leading to CNVs, non-homologous end joining (NHEJ) may especially produce them in sub-telomeric regions [34], and may have led to relatively more recent changes [67]. We did not have sufficient statistical power to assess relative contributions of deletions in different chromosomal locations. Future studies, however, might benefit from doing so.

Naturally, we do not suggest that *every* rare CNV deletion affects intellectual functioning. Almost certainly, many do not. But again, were it the case that only very small proportion of deletions did so, there would exist only a very weak association between FSIQ and a composite of deletions. As this is not what we observed, it seems very likely that a meaningful, substantial proportion of CNVs are associated with intellectual functioning.

As emphasized in the introduction, a primary impetus for examining associations between rare CNV deletions and intellectual functioning is the theoretical perspective that argues that genetic variation in psychometric intelligence is largely due to the existence of individually rare but, at a genome-wide level, ubiquitous deleterious variants—mutations that are selected against—rather than the existence of recently arisen, positively selected variants [23]. Our results are consistent with this proposal. Although CNV deletions may constitute a major form of deleterious variants, this theoretical perspective also expects that some mutations at the single nucleoside level affect intelligence as well. Our findings are also consistent with Miller's theory [68] that mate selection based on intelligence may provide a mechanism to optimize “good genes” in offspring (but see also [69]).

We found that rare deletion length predicted intelligence in our Anglo/White sample, but not in our Other Ethnicity sample. These results are consistent with previous findings that the heritability of g is greater in children and adolescents coming from high SES backgrounds [52], [53] (though see [70] for a recent contrary report based on a large and diverse adult sample). The relatively enriched environments of high SES families may potentiate the expression of genetic make-ups promoting high intellectual performance. As we found no difference across ethnicities in residual variance in intelligence once deletion length was controlled, it remains possible that total absolute variance in FSIQ associated with non-genetic factors does not differ across groups. It should also be noted that ethnicity is an imperfect indicator of parental SES, which was not available to us, and that the sociocultural aspects of minority status may impact neurodevelopment [71], [72].

### Limitations

There are two sets of important limitations of the current study. The first relates to our sample. Though our results have clear implications for the origins of comorbidity among externalizing disorders, and the potential to help account for the genetic vulnerability associated with alcohol abuse, these issues can be best pursued in future studies that provide diagnoses of all possible comorbid disorders and include a healthy control group. The lack of a significant association between alcohol dependence and intelligence and the aggregate CNV measures may be due to the fact that the sample was limited to only individuals with alcohol dependence. In light of the numerous failures of replication in history of molecular genetic studies of intelligence [73], efforts at replication and extension would also benefit from a larger sample size.

The second set of important limitations concerns our assessment of CNVs. Though we believe we have achieved valid and reliable estimates, it is undoubtedly the case that we did not capture all rare CNVs in our participants' genomes. Some smaller CNVs and those in regions not as well mapped by the reference genome may have gone undetected. Whether inclusion of such CNVs would strengthen or weaken our findings is unknown. We have made two major assumptions in the quantification of CNVs, which should be systematically evaluated in larger studies. First, we arbitrarily defined rare CNVs as those occurring in 5% or fewer of our participants. However, use of 1% and 3% cutoffs led to similar effect sizes. An important question for future research is how different phenotypes relate to CNVs occurring at different population frequencies. Possibly, variations in intelligence within the normal range are subject to less selection pressure than phenotypic variation associated with debilitating disorders such as schizophrenia or autism, and hence, associated with relatively more common variants.

As more common CNVs might be less deleterious [35], a more stringent cutoff may have produced stronger results. Studies linking total rare CNVs to schizophrenia have used a 1% frequency criterion [41]. Second, we eliminated approximately 8% of our sample due to extreme CNV total values, though we used standard procedures to do so. Outliers can obviously have a strong effect on effect size estimates, and continued advances in analytic quality control will be important.
